# The philosophy of pharmaceutical regulation—Paternalism or freedom of choice?

**DOI:** 10.3389/fmed.2023.1264021

**Published:** 2023-10-27

**Authors:** Jörg Mahlich, Sybille Riou, Matthieu Verry

**Affiliations:** ^1^Miltenyi Biomedicine, Bergisch Gladbach, Germany; ^2^Düsseldorf Institute for Competition Economics (DICE), Heinrich-Heine-University Düsseldorf, Düsseldorf, Germany; ^3^Écologie Responsable, Paris, France

**Keywords:** liberalism, regulation, uncertainty, Hegel, Smith, decision making

## Abstract

When assessing the value of new drugs regulatory authorities across the world frequently make different decisions even though their decisions are based on the same evidence package. In this perspective we argue that even in today’s world regulatory and medical decision making is framed by conflicting philosophical schools of thought, namely the liberal tradition of the Anglo Saxon countries pioneered by the Scotsman Adam Smith and the continental European tradition of paternalism that roots back to the German philosopher Georg Friedrich Hegel. We outline the basics of these two philosophical theories and show that countries following the liberal tradition are more reluctant to reject new drugs due to weak evidence. Instead, they leave decisions to a greater extend to those who are affected, namely patients and their caregivers.

## 1. Introduction

Regulatory decision makers are supposed to act for the benefit of the public by taking corrective action when markets fail to allocate resources efficiently. This would assume that regulators are aware of public preferences when they balance trade-offs. Several trade-offs are relevant when it comes to drug marketing approval.

(1) Weighing of endpoints: The first is how endpoints of clinical studies are weighted and to find the right balance of harm and benefit. E.g., should a therapy that marginally prolonged life on the expense of quality of life be approved. Or imagine a situation where all endpoints except one are worse, how much weight should this endpoint get?
(2) Acceptance of uncertainty: How much uncertainty about efficacy and safety is acceptable? In this context it is helpful to distinguish between Knightian uncertainty which is a lack of any quantifiable knowledge about some possible occurrence, as opposed to the presence of quantifiable risk (1). Here regulators can do two error types in the drug approval decision, according to Manski (2). A type I error is when a new drug is approved based on preliminary data although it is actually inferior to the comparator. A type II error happens when better drugs do not receive approval because at the time of drug assessment, the data are still immature and do not prove superiority. This is a trade-off between fast access that allows patients to potentially benefit early from a new drug and potential damages.
(3) Valuation of Risk: Regarding risky situation i.e., when outcomes are associated with a quantifiable probability, the question is how to value risk. The term “value of hope” for instance refers to risk seeking individuals who prefer outcomes that are associated with a high variance because they value the potential gains more than potential losses (3). A drug that offers on average a lower life expectancy than the established standard of care and is even riskier in that the variance of life expectancy is higher might still be preferred by patients because the high variance offers sufficient opportunities that are of value for patients.

All those trade-offs can, in principle, be addressed by utilizing patient preference studies such as discrete-choice-experiments (4). Currently authorities such as the FDA developing guidelines for patient-focused drug development (PFDD) that should ensure that data relating to patient and caregiver experience are heard in regulatory decision making (5). The European counterpart of PFDD is PREFER (Patient Preferences in Benefit-Risk Assessments during the Drug Life Cycle), a public-private collaborative research project under the Innovative Medicines Initiative (IMI) with an involvement of the EMA and other relevant stakeholders (6). However, two problems arise: even if a regulatory decision makers act as agents of the people as the principal and try to serve the interest of the public (“benevolent regulator”), it is impossible in practice to gather sufficient information and determine demand in heterogeneous societies. This “knowledge problem” was first identified by Hayek, prompted by Tocqueville and Lord Acton (7) and can explain why the notion of a planned economy is nothing more than “the road to servitude”–to quote the name of his polemical essay published in 1944 before the fall of the Berlin Wall and the collapse of the Soviet Union. Because what is known by a single agent is only a small fraction of the sum total of knowledge held by all members of society. Furthermore, principal-agent problems can occur when decision makers follow their own interest that conflicts with the interests of the principal (so called private interest hypothesis) (8). According to Quirk (9), the FDA staff is primarily interested in a good reputation of their work and attempting to avoid type I errors which implies a focus on safety rather than on the rapid access of new drugs. Another graphic example is the interplay of industry regulation and contributions to political parties by the pharmaceutical industry (10). Powerful firms can shape the environment they are operating in, an assertion first made by Nobel Laureate Stigler (11) in his theory of economic regulation: “… as a rule, regulation is acquired by the industry and is designed and operated primarily for its benefit.” It has been shown that regulation in the pharmaceutical industry constitute entry barriers for small firms for the benefit of large firms that can increase their market power on expense of smaller firms (12, 13). Thus, regulators either deliberately make decision that are in their own interests rather in that of the public or, even if they try to make good decisions, they are not able to do so because of the knowledge problem.

## 2. Philosophical foundation

Against this background we argue that US and continental Europe have two diverging traditions that shapes the discretionary power of regulators. One is the liberal school of thought pioneered by Adam Smith who stated that self-seeking individuals are guided by an “invisible hand.” Even free and self-interested individuals unintentionally advance the interest of the society. In a famous quote Smith has described the invisible hand as follows: “It is not from the benevolence of the butcher, the brewer, or the baker, that we expect our dinner, but from their regard to their own interest. We address ourselves, not to their humanity but to their self-love, and never talk to them of our own necessities but of their advantages” (14). The continental European opposite of this view was raised by Georg Wilhelm Friedrich Hegel who saw the “sphere of liberty as the whole state, with freedom not so much an individual’s right, but rather, a result of human reason” (15, 16). In his logic, it was the benevolent state that is the basis of a prosperous society: “the constitution of the political state brings together in a unity the sense of the importance of the whole or universal good along with the freedom of particularity of individual pursuits” (17). According to Hegel, the population does not have the “consciousness” to be directly involved in decisions opposed to the state (17). This paradigm between the individual and the State, in the light of these two diametrically opposed philosophers, pushes human to reinvent himself in an ambivalence that characterizes him. Philosophers have always explored the question of freedom, notably Aristotle, who questions the volunteer and involuntary in his book III *Nicomachean Ethics*. Then, several theories have been issued, including two complementary readings from different horizons by Saint Thomas Aquinas in the *Summa Theologica* and Paul Ricoeur in his article “Liberté” in the *Encyclopaedia Universalis*. Ricoeur’s relevant question is the capable subject, and he seeks to understand the human subject from what he does within the prism of historical consciousness, hermeneutics as well as ethics. Thomas, on the other hand, is a Dominican theologian who seeks to choose better in order to come closer to God.

In a more recent context, the philosophy of science is leading us to rethink health via autonomy. Among others, the writings of Flanigan denounce the presence of paternalism and infantilization in healthcare (18, 19). These reflections have turned the entire liberal philosophical movement on its head, bringing a crucial breath of freedom to bear on issues that affect the individual at the deepest level of his or her being. Philosophy makes it possible to question the importance of reasoning and consenting in making choices of which are *intra* and not just *extra*.

Freedom and health have also been studied by Friedman, one of the fathers of health liberalism. Indeed, he asserts that the lack of freedom through the “quality” criteria of peers in the field of medical training does not make it possible to achieve the correct equilibrium point. He observes that the 1929 crisis created unemployment and lower incomes, opening up the medical field to a greater number of people, particularly in the USA. The influx of these new, additional foreign doctors helped to strengthen organization and quality requirements through the advent of new practices. In addition, he supports his thesis with other arguments, such as the loss of time spent on procedures that can be delegated, the incentive to find ways around restrictions, and the fact that the performance of an examination underwent twenty or thirty years ago does not guarantee its present quality. Moreover, according Friedman, the absence of freedom in the medical field reduces the possibility of experimentation and thus of acquiring the related knowledge, which “reduces both the quantity and quality of medical practice, diminished the number of opportunities open to those who wanted to be doctors and were forced to embrace professions they considered less attractive, forced the public to pay more for less satisfactory medical services, and retarded the technological development of both medicine itself and the organization of its practice” (20). As a result, this can be a heavy social blow for on the one hand the public and on the other hand individuals who wish to practice medicine. As such, Friedman argues that there are two solutions for developing medicine: the end of central government planning (professional monopolies), and the response of the market, which has the tolerance (diversity and ability to use a vast body of knowledge) allowing consumers to make their own choice.

## 3. Differences between FDA and EMA

To illustrate that both Hegel and Smith’s schools of thought still have an impact on today’s regulatory decision making, we compare the European decision maker European Medicines Agency (EMA) with the Food and Drug Administration (FDA) from the US assuming that FDA has a more liberal stand while EMA is influenced by the continental European heritage of paternalism.

Among the three trade-offs identified in the introduction, the acceptance of uncertainty is a very relevant one. In regulatory decision making this mainly reflects acceptance of data that were not collected by means of the “gold standard” i.e., randomized clinical trials (RCT). Those studies are potentially subject to confounding which is difficult to adjust for in indirect comparisons (21). In such a situation, some uncertainties about efficacy and side effects will remain. To accept or not accept those uncertainties is not a scientific problem but purely based on subjective preferences of the relevant actors involved. According to Tafuri et al. (22), the FDA shows a greater willingness to approve uncertainties related to phase-II single-arm trials (SAT) vs. EMA. This claim motivated the literature review in which we analyze the acceptance by regulators of SAT. While both the FDA and EMA are aligned in principle on the use of single-arm studies when there is no effective treatment in a given indication or when a RCT is not feasible, the rejection rates of SAT submissions differ significantly between the FDA and EMA as Table 1 shows. While SAT are rejected in 21% of the cases by EMA, the respective rate is only 2% for FDA between 2005 and 2017, with all drugs rejected by the EMA were approved by the FDA (23). An older study comes to similar conclusion for the period 1999–2014 (24). Among SAT-based applications submitted to both the FDA and EMA (*n* = 44), the FDA rejected only one out of 44 (2%) while EMA rejected nine out of those 44 (20%) (24). In addition, FDA tends to grant more accelerated approvals than EMA’s conditional authorizations (25).

**TABLE 1:** Difference in approvals of single-arm trials (SAT), real-world evidence (RWE) and time to review between the EMA and FDA.

Criteria	Time period	EMA	FDA	References
SAT approval in% (*N*)	2005–2017	79% (27/34)	98% (40/41)	(23)
SAT approval in% (*N*)	1999–2014	80% (35/44)	98% (43/44)	(24)
Conditional/accelerated approval of SAT in% (*N*)	2005–2017	24% (8/34)	56% (23/41)	(23)
RWE approval in% (*N*)	2005–2017	95% (17/18)	100% (27/27)	(23)

EMA, European Medicines Agency; FDA, Food and Drug Administration; SAT, single-arm trial; RWE, real-world evidence.

The acceptance rate of real-world evidence (RWE) is another indicator of the willingness to accept uncertainty. As shown in Table 1, 95% of SAT with RWE (undefined external controls) were approved by EMA and 100% by FDA (23).

## 4. Differences among HTA bodies

We now examine if cultural differences prevail at the health technology assessment (HTA) level as well. For this, we classify the biggest HTA agencies according to their cultural heritage. National Institute for Health and Care Excellence (NICE) of UK, Canadian Agency for Drugs and Technologies in Health (CADTH) of Canada and Pharmaceutical Benefits Advisory Committee (PBAC) of Australia belong to the more liberal Anglo-Saxon tradition due to their colonial history. On the other hand, Germany’s Gemeinsamer Bundesausschuss (GBA)/Institut für Qualität und Wirtschaftlichkeit im Gesundheitswesen (IQWiG), and Haute Autorité de santé (HAS) of France can be classified into the continental European tradition. We examine if we can observe different attitudes to the tolerance of uncertainties by checking the acceptance rate of SAT for benefit assessments. Note, that as this stage drugs have already clearance from the EMA. Table 2 reports the share of positive (including positive with restrictions) recommendations of single-arm trials submissions.

**TABLE 2:** Share of positive (including positive with restrictions) recommendations of single-arm trials submissions.

			Philosophical tradition					
			**Continental**		**Anglo-Saxon**			
**Time period**	***N***	**With/out RWE**	**GBA/IQWIG**	**HAS**	**CADTH**	**NICE**	**PBAC**	**References**
2010–2015	27	Without	17% (1/6)	NA	69% (11/16)	60% (3/5)	NA	(26)
2011–2019	186	With	70% (14/20)	46% (12/26)	72% (13/18)	90% (19/21)	50% (11/22)	(27)
		Without	42% (5/12)	32% (13/41)	60% (9/15)	33% (1/3)	63% (5/8)	
		Total	59% (19/32)	37% (25/67)	67% (22/33)	83% (20/25)	53% (16/30)	

CADTH, Canadian Agency for Drugs and Technologies in Health; GBA, Gemeinsamer Bundesausschuss; HAS, Haute Autorité de santé; IQWiG, Institut für Qualität und Wirtschaftlichkeit im Gesundheitswesen; NICE, National Institute for Health and Care Excellence; PBAC, Pharmaceutical Benefits Advisory Committee; RWE, real-world evidence.

Here results are less clear. While NICE and CADTH tend to have higher acceptance rates than the continental European counterparts of HAS and GBA, Australian’s PBAC is a kind of an outlier with acceptance rates closer to the ones observed at HAS and GBA/IQWIG. The overall trend toward higher acceptance of uncertainty in the Anglo-Saxon countries, however, is still intact. It also worth to mention that both in France and Germany positive recommendations of SAT are not so much based on the acceptance of the SAT based indirect comparisons but based on other institutional features of the HTA system (28). For instance, orphan drugs in Germany receive a positive recommendation by law regardless of their submitted evidence package (29, 30).

## 5. Conclusion

We showed that there are two conflicting philosophical schools of thoughts that still have an impact on today’s practice of medical decision making. The Hegelian tradition has resulted in a paternalism of authorities and leaves less room for individual choices. In contrast, the liberal Smithian tradition focus on the individuum and argues that regulators either do not possess the relevant information to make good choices, or even worse, make decision that are only in their own private interests. As this perspective article is intended to be hypothesis-generating we leave it to future research to rigorously test the association between philosophical heritage and regulatory outcomes. Such research needs to account for potential confounders such as therapeutic indication, or the study design of the submitted SATs. Another potentially fruitful path would be to examine the impact of the withdrawal of the UK from the European Union on EMA’s decision making. Ceteris paribus, we would expect a shift toward a more paternalistic approach as UK’s Medicines and Healthcare products Regulatory Agency (MRHA) liberal voice is not part of the EMA anymore.

## Data availability statement

The original contributions presented in this study are included in the article/supplementary material, further inquiries can be directed to the corresponding author.

## Author contributions

JM: Conceptualization, Writing – original draft. SR: Writing – original draft. MV: Writing – original draft.
